# Supplementary material for: Behavioral age-detection in individuals reconstructs minute-scale developmental transcriptomics
Source: bioRxiv. 2025 Nov 2:2025.11.01.686022. Preprint. [Version 1] doi: 10.1101/2025.11.01.686022 (PMC12636446; doi:10.1101/2025.11.01.686022)
Supplement: Supplement 1 [file NIHPP2025.11.01.686022v1-supplement-1.pdf]

bioRxiv preprint doi: https://doi.org/10.1101/2025.11.01.686022; this version posted November 2, 2025. The copyright holder for this preprint (which was not certified by peer review) is the author/funder, who has granted bioRxiv a license to display the preprint in perpetuity. It is made available under aCC-BY 4.0 International license.

Figure S1

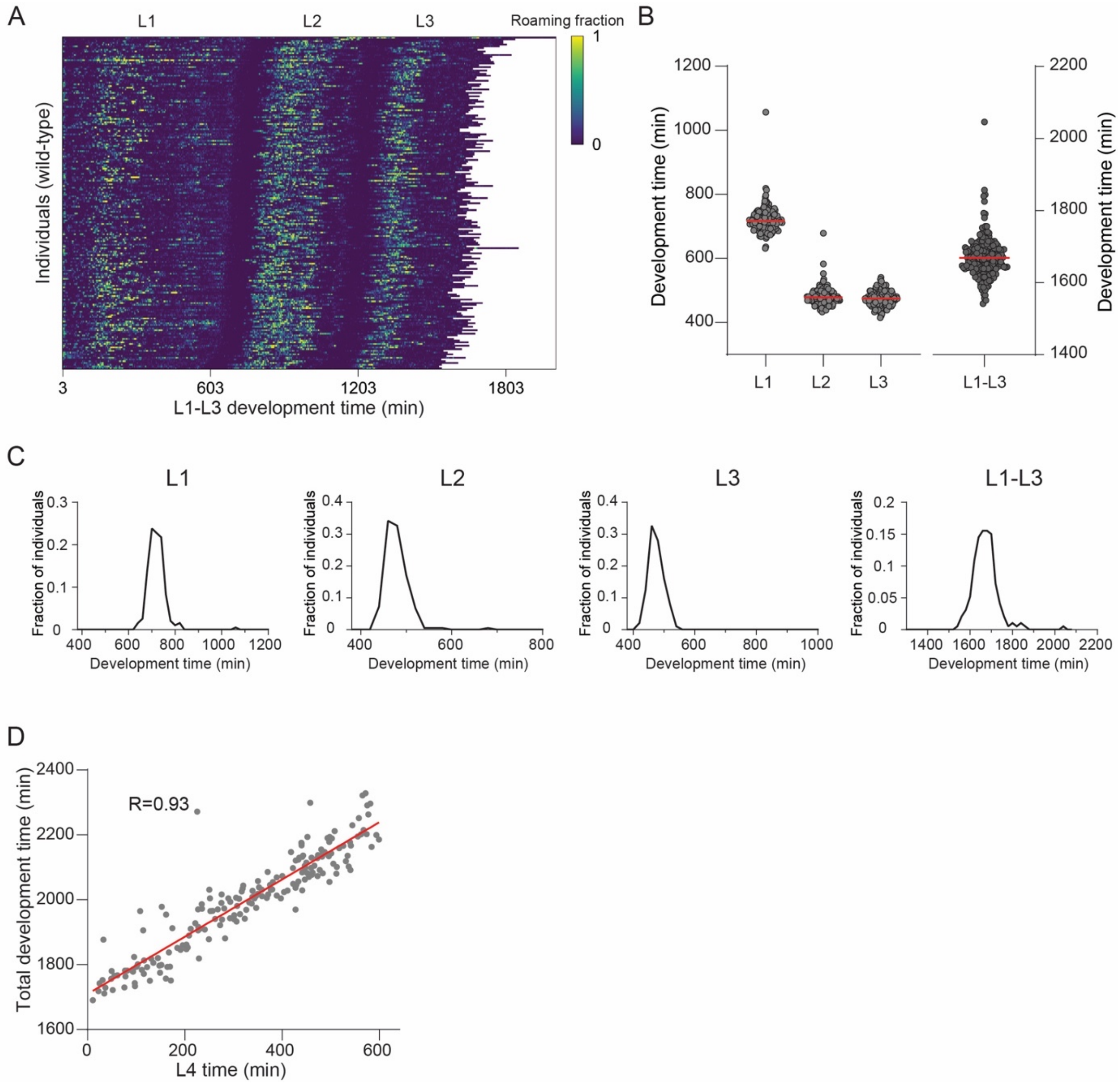

Figure S1. Inter-individual variation in development time

(A) Heatmap of roaming activity across the L1, L2, and L3 developmental stages in wild-type individuals (n = 193). Each row represents a single individual. Color indicates the fraction of time spent roaming in each of the 3-minute time-bins across the individual's trajectory (see Methods). Heatmap is sorted based on L1 duration. **(B)** Plot shows the duration of each developmental stage and total time of the L1-L3 stages of wild-type individuals. Each dot represents a single individual. Red line marks the average duration. **(C)** Distributions of stage durations of each larval stage across individuals as in (B). **(D)** Correlation (Pearson) between the duration in the L4 stage and the total developmental time since hatching of wild-type individuals.

bioRxiv preprint doi: https://doi.org/10.1101/2025.11.01.686022; this version posted November 2, 2025. The copyright holder for this preprint (which was not certified by peer review) is the author/funder, who has granted bioRxiv a license to display the preprint in perpetuity. It is made available under aCC-BY 4.0 International license.

Figure S2

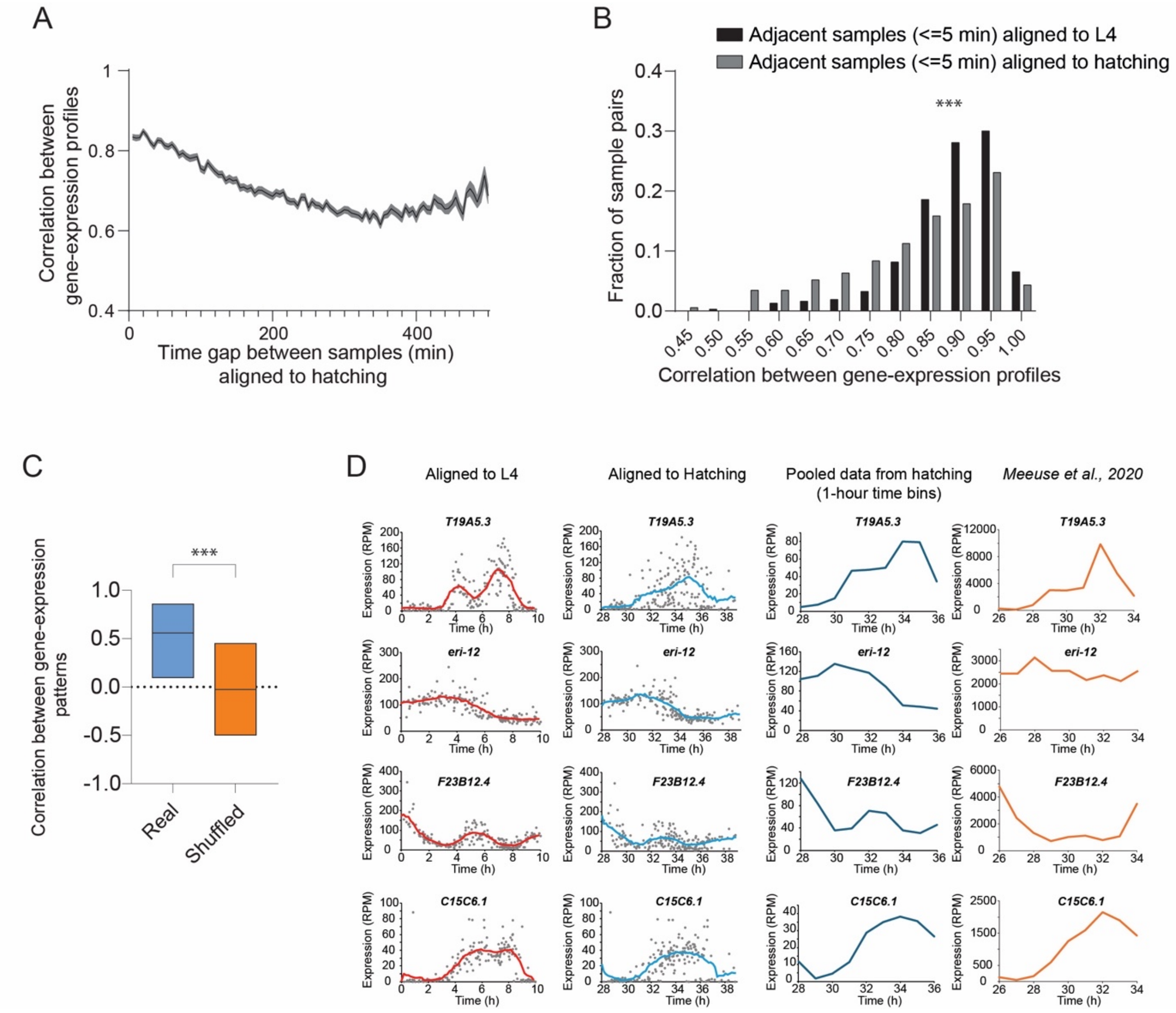

Figure S2. Similarity in transcriptional profiles of adjacent individuals and comparison to pooled dataset

(A) Average correlation between gene-expression states of pairs of individuals with a specific time gap between their developmental age. Shaded grey area represents standard error of the mean. Developmental age is aligned to the hatching time. **(B)** Comparison of distribution of correlation values between gene-expression states of pairs of individuals collected within 5 minutes of each other, based on L4 time-alignment (black) relative to time-alignment to hatching (grey). *** P-value<0.001, Kolmogorov-Smirnov test. **(C)** Correlation (Pearson) between temporal gene expression trajectories in our dataset, artificially pooled in each 1 hour time-bin (n=9740, see Methods) and a previously generated hourly pooled dataset (*Meeuse et al*. 2020) (Blue). Real correlations are compared to random correlations between shuffled datasets (orange). Boxes represent the variation (IQR) of the respective distributions. **** P-value<0.001, unpaired t-test. **(D)** Examples of gene expression

bioRxiv preprint doi: https://doi.org/10.1101/2025.11.01.686022; this version posted November 2, 2025. The copyright holder for this preprint (which was not certified by peer review) is the author/funder, who has granted bioRxiv a license to display the preprint in perpetuity. It is made available under aCC-BY 4.0 International license.

profiles over time reconstructed from individuals aligned to L4 and hatching, as well as artificially pooled in each 1-hour time-bin in our dataset and in the hourly pooled dataset previously generated in *Meeuse et al*. (2020). Comparison focuses on developmental hours 28–36 in our dataset and hours 26–34 in *Meeuse et al.*, due to differences in growth temperature.

bioRxiv preprint doi: https://doi.org/10.1101/2025.11.01.686022; this version posted November 2, 2025. The copyright holder for this preprint (which was not certified by peer review) is the author/funder, who has granted bioRxiv a license to display the preprint in perpetuity. It is made available under aCC-BY 4.0 International license.

Figure S3

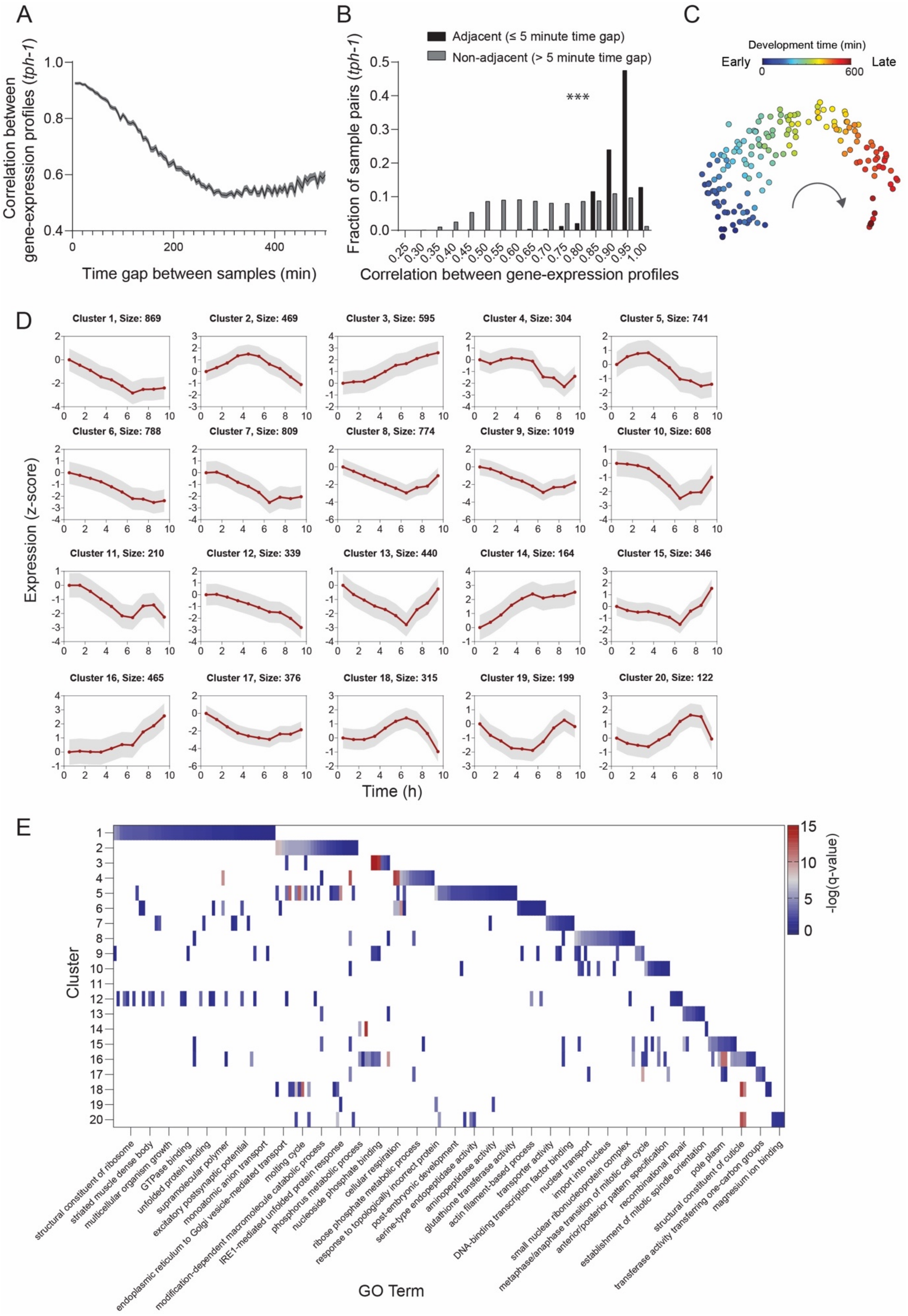

bioRxiv preprint doi: https://doi.org/10.1101/2025.11.01.686022; this version posted November 2, 2025. The copyright holder for this preprint (which was not certified by peer review) is the author/funder, who has granted bioRxiv a license to display the preprint in perpetuity. It is made available under aCC-BY 4.0 International license.

Figure S3. Time-reconstruction of temporal gene-expression patterns in *tph-1* serotonin-deficient individuals

(A) Average correlation between gene-expression states of pairs of *tph-1* individuals with a specific time gap between their developmental age. Shaded grey area represents standard error of the mean. Developmental age is aligned to the start of the L4 stage. **(B)** Comparison of distributions of correlation values between gene-expression states of pairs of *tph-1* individuals separated by less than or equal to 5 minutes in their developmental age (black) relative to pairs of individuals separated by more than 5 minutes (grey). *** P-value<0.001, Kolmogorov-Smirnov test. **(C)** t-distributed Stochastic Neighbor Embedding (t-SNE) visualization of all *tph-1* samples (n=164) based on gene expression profiles. Each point represents a single individual. Each single-individual sample is color-coded based on the identified developmental age. **(D)** Average temporal expression within each of the 20 gene clusters in Fig. 5B (smoothed using a window size of 1.5 hours, intervals of 0.49). Grey shaded area indicates the standard deviation. **(E)** Heatmap denotes Gene Ontology (GO) enrichment analysis results for each gene cluster generated from the *tph-1* dataset. Clusters are listed on the y-axis. Color code indicates the significance of enrichment for all significantly enriched GO biological functions (q-value<0.01). GO biological functions indicated by text in the figure represent only a partial list. Full list is in Supp Table S4.

bioRxiv preprint doi: https://doi.org/10.1101/2025.11.01.686022; this version posted November 2, 2025. The copyright holder for this preprint (which was not certified by peer review) is the author/funder, who has granted bioRxiv a license to display the preprint in perpetuity. It is made available under aCC-BY 4.0 International license.

Figure S4

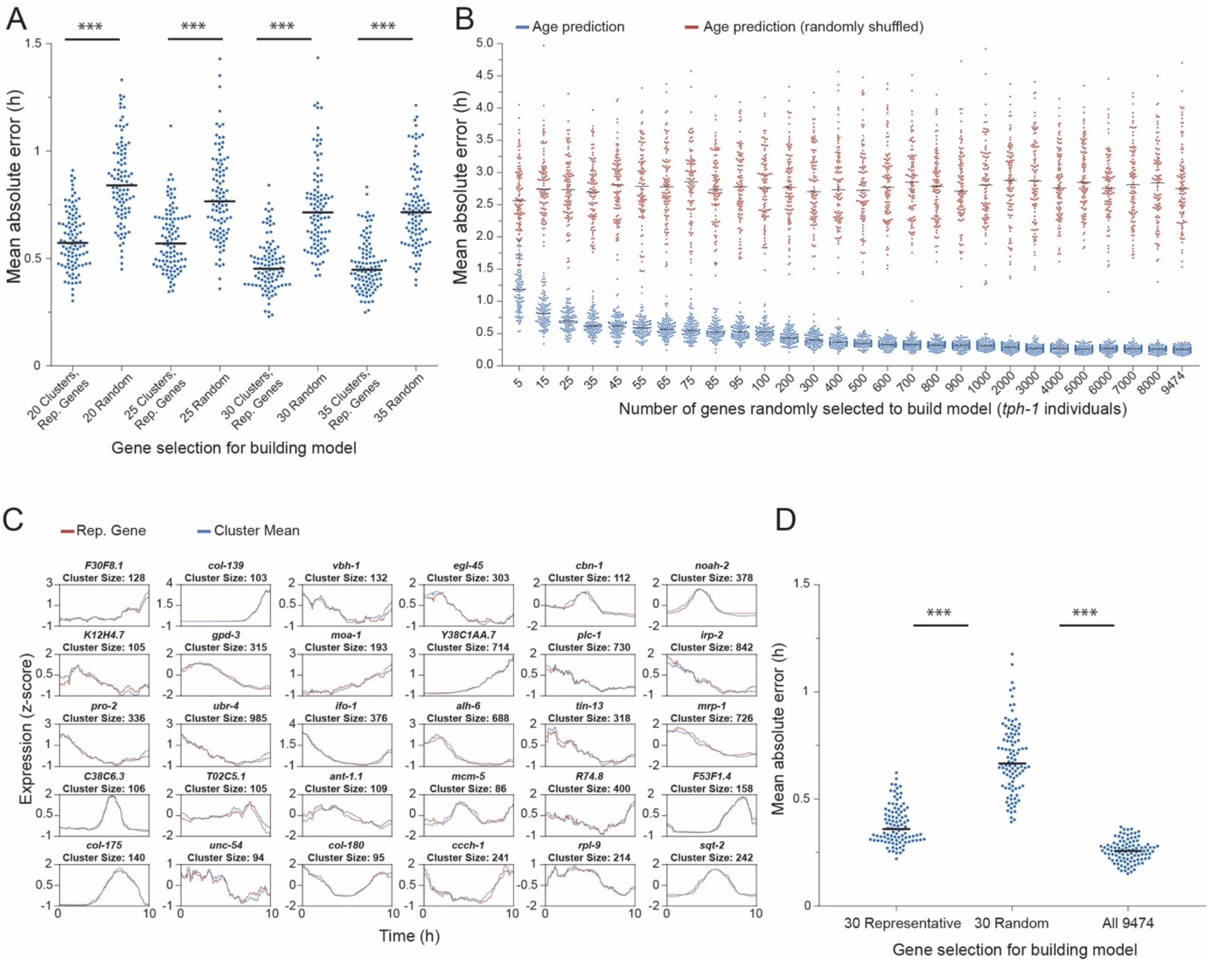

Figure S4. Developmental age prediction in wild-type and *tph-1* individuals

(A) Comparison of precision in developmental-age prediction using representative genes of wild-type individuals that correspond to variable number of generated clusters. Each dot represents a single trial. Black lines mark the average precision across trials. *** P-value<0.001 (Mann Whitney U test) for the difference in average error, compared to predictions using a randomly shuffled dataset. **(B)** Shown is the prediction precision (average absolute error) of *tph-1* individuals developmental-age by a neural network model generated using random subsets of genes of different sizes (5 to total set of 9474 active genes in both genotypes) (see Methods). **(C)** Representative genes (red) of distinct clusters (see Methods) generated from the *tph-1* individuals dataset. Each representative gene was identified as having the highest correlation (Pearson) to the mean expression of each cluster (blue). **(D)** The prediction precision of neural network models generated using the 30 representative genes as in (C), compared to a random selection of 30 genes, and to the total set of

bioRxiv preprint doi: https://doi.org/10.1101/2025.11.01.686022; this version posted November 2, 2025. The copyright holder for this preprint (which was not certified by peer review) is the author/funder, who has granted bioRxiv a license to display the preprint in perpetuity. It is made available under aCC-BY 4.0 International license.

9474 genes shared across genotypes (100 trials). Each dot represents a single trial. Black lines mark the average precision across trials. ***P-value < 0.001, Mann-Whitney U Test.
